# The psychological compensation effect: perceived digital exclusion, perceived lack of control, and their impact on consumption behaviors of elderly tourists

**DOI:** 10.3389/fpsyg.2026.1764922

**Published:** 2026-05-04

**Authors:** Jinna Shi, Yuxin Li, Weiwei Deng

**Affiliations:** School of Tourism and Culture Industry, Sichuan Tourism University, Chengdu, China

**Keywords:** compensatory consumption behavior, perceived digital exclusion, perceived loss of control, self-affirmation, social cognitive theory

## Abstract

The deep integration of digital technology in the tourism industry has made perceived digital exclusion a significant challenge affecting the travel experiences of elderly tourists. However, the specific psychological pathways and boundary conditions through which perceived digital exclusion influences their consumer behavior remain unclear. Grounded in social cognitive theory, this study proposes a “perceived digital exclusion–compensatory consumption” framework, focusing on the mediating role of perceived loss of control and the moderating role of self-affirmation. A questionnaire survey was conducted with 328 elderly Chinese tourists. The findings reveal that: (1) perceived digital exclusion is positively associated with compensatory consumption behaviors among elderly tourists; (2) perceived loss of control mediates the relationship between perceived digital exclusion and compensatory consumption; and (3) self-affirmation negatively moderates the effects of both perceived digital exclusion and perceived loss of control on compensatory consumption. Accordingly, this study clarifies that perceived loss of control serves as a key psychological mechanism linking perceived digital exclusion to compensatory consumption, and reveals the buffering effect of self-affirmation on this mechanism, thereby delineating its psychological boundaries. The findings suggest that in addressing the digital divide, tourism service providers and managers should look beyond technical access and pay greater attention to supporting the psychological needs of elderly tourists. Mitigating the negative consequences of perceived digital exclusion—by enhancing older tourists’ sense of control and providing effective non-digital alternatives—can foster positive market behavior and contribute to a more inclusive tourism environment.

## 1 Introduction

Understanding public attitudes toward digital facilities is essential for research on the digital divide. To this day, the gap in perceived digital exclusion (PDE) remains substantial (Wilson-Menzfeld et al., 2025), and individuals continue to be marginalized due to their inability to use digital technology effectively (Yao et al., 2025). As a multifaceted sociotechnical phenomenon, PDE has been diversely conceptualized across scholarly debates (Vassilakopoulou and Hustad, 2023). Recent literature identifies three levels of digital exclusion—digital access, digital use, and digital confidence or skills (Wilson-Menzfeld et al., 2025).

The extent of these gaps among older populations is well documented. Statistically, while internet penetration continues to rise across age groups, a clear age-based digital divide persists within the older adult population itself. Studies consistently report that internet and smartphone use among individuals in their late 70s and beyond is substantially lower—often by a margin of 20 to 30 percentage points—compared to those in their early 60s, suggesting that chronological age remains a meaningful differentiator even among older adults. This pattern has been observed across multiple national surveys and regional studies, though exact figures vary by country and sample composition. Beyond basic access, limitations in technological skills follow a similar yet more nuanced pattern. Research on digital skills often distinguishes between operational skills (e.g., using basic functions), information navigation skills (e.g., searching and evaluating online content), and strategic skills (e.g., using digital tools to achieve complex goals). Within this classification, older adults are disproportionately concentrated at the lower end. Many can manage routine tasks such as making calls or reading messages but encounter significant difficulty with activities requiring judgment—such as identifying reliable information, adjusting privacy settings, or completing multi-step transactions.

Qualitative studies further illustrate the nature of these difficulties. Accounts from older individuals frequently describe how unfamiliar interface designs, fear of making irreversible mistakes, and the absence of timely assistance lead them to withdraw from digital interactions altogether, even when devices are available. This layered pattern—from access to skills, and from skills to psychological withdrawal—suggests that digital engagement among older adults is less a binary state than a continuum shaped by age, prior experience, and the availability of contextual support.

These technical and skill-based barriers are often intertwined with broader physiological, psychological, and social factors, including limitations in flexibility and vision, confidence and self-efficacy (Wilson et al., 2023), fear, trust, and security issues (Age UK, 2021), as well as a lack of social capital (Tsai et al., 2015).

Although the pervasiveness of digital life and the daily impact of PDE on individuals across society are now widely acknowledged, existing research in this field has primarily focused on specific, smaller populations. These include mental health service users (Greer et al., 2019), immigrants (Bastick and Mallet-Garcia, 2022), as well as the negative effects on older adults’ mental health (Chen S. X. et al., 2024) and social participation (Seifert et al., 2021). Nevertheless, how PDE specifically influences the decision-making mechanisms underlying travel consumption among older adults remains underexplored. This gap is particularly salient as two long-term trends converge. The global population over 60 is expected to reach 2.1 billion by 2050, making older adults an increasingly central segment in tourism markets. At the same time, the tourism industry continues to embed digital technologies into nearly every stage of travel—reservations, navigation, payment, and experience sharing. What seems like seamless convenience for many can become a barrier for those who are less digitally prepared. When older tourists repeatedly encounter such barriers, their participation may be limited, and unintended psychological responses—such as compensatory consumption patterns aimed at regaining a sense of control—may emerge. Left unexamined, these dynamics could lead to negative outcomes for older consumers, who may engage in irrational spending, and for service providers, who may miss opportunities to build genuinely inclusive systems. Understanding how PDE translates into consumption behavior is therefore not just about filling a research gap; it is a prerequisite for designing interventions, service processes, and policy supports that align with the psychological realities of aging populations.

In tourism settings, digital technologies—such as smart booking systems, electronic guides, and mobile payments—can become sources of implicit exclusion for older adults. Due to declining technological adaptability and reduced cognitive flexibility, older tourists often encounter difficulties with online reservations and mobile payment systems, compelling them to resort to traditional offline service channels (Seifert et al., 2021). Such limitations not only restrict their equitable access to tourism resources but may also induce irrational consumption behaviors through specific psychological mechanisms (Li et al., 2018).

Against the backdrop of accelerated global aging and the deep penetration of digital technologies, examining compensatory consumption behavior (CCB) among older tourists (OTs) in contexts of PDE contributes to enhancing global understanding of the digital divide and its societal impacts. To uncover the intrinsic psychological mechanisms through which PDE influences the travel consumption behavior of older adults, this study adopts social cognitive theory (SCT) (Kursan Milaković, 2021) as its foundational framework and employs compensatory consumption theory (Wang and Tian, 2025) to address the following research questions:

*RQ1*: Does PDE lead to CCB among OTs during travel?

*RQ2*: If so, how is this phenomenon generated?

*RQ3*: Can CCB be reduced through self-affirmation (SA)?

## 2 Literature review and hypotheses development

### 2.1 The impact of perceived digital exclusion on compensatory consumption behavior in senior tourism

Research on senior tourism has encompassed various dimensions, including travel motivation, decision-making mechanisms, active aging, value co-creation, tourism consumption, and market analysis (Fan et al., 2025). However, insufficient attention has been paid to the applicability of digital technologies among this population. As tourism services become increasingly digitalized, older adults are often marginalized as “digital refugees” due to difficulties in using technology, experiencing systematic exclusion in areas such as booking, payment, and navigation (Chen S. X. et al., 2024). This form of exclusion is defined as social marginalization resulting from a lack of digital literacy (Yao et al., 2025), representing a new manifestation of traditional social exclusion in the information age (Van Dijk, 2020).

Social exclusion is a prevalent negative social phenomenon in which individuals or groups are rejected by others or social communities, thereby threatening their needs for belonging and interpersonal relationships (Nezlek et al., 2012). In the wake of the digital wave, this concept has given rise to a new form—perceived digital exclusion (PDE). PDE refers to the sense of exclusion individuals experience due to their inability to use digital technologies effectively. As digital technologies become deeply embedded in everyday life, older adults—hindered by technological barriers—increasingly struggle to access smart services and are often marginalized as “digital refugees” (Chen S. X. et al., 2024). In tourism settings, the social exclusion encountered by older adults demonstrates multidimensional and dynamic features, encompassing social relationships, service facility accessibility, socio-cultural exclusion, and PDE (Seifert et al., 2021).

Digital technologies are now extensively integrated into the entire tourism service process, offering notable benefits such as convenience, efficiency, and the elimination of spatial and temporal limitations. However, for older adults who are digitally marginalized, smartphones and the internet have become significant barriers to participation. Lacking the skills to use new technologies—including online ticket booking, service purchases, and smart ride-hailing—they are often excluded from digital service systems. As a result, they are compelled to rely on traditional offline channels, such as booking tickets at physical counters, paying with cash, or using paper maps, or to seek help from younger generations—for instance, by asking their children or grandchildren to make online reservations or navigate using smartphone apps (Yao et al., 2025).

The theory of compensatory consumption originated from the study of psychological adaptation mechanisms. Gronmo (1988) pioneered the integration of compensatory mechanisms into consumption research, arguing that consumption acts as a substitute means of fulfilling self-esteem or self-realization when systematic imbalances arise between needs and behaviors. Wang and Tian (2025) further elaborated on the operational pathways, suggesting that compensatory consumption involves both an unconscious reaction to identity threats and a deliberate effort to construct an ideal psychological state through consumption. This expanded the scope of compensation from tangible products to symbolic consumption experiences. Essentially, compensatory consumption functions as a substitute mechanism to compensate for psychological deficits or threats through purchasing behavior, rather than being motivated by functional value alone.

Research has shown that social exclusion impairs fundamental human needs—such as belonging, control, self-esteem, and meaningful existence—elicits negative emotions including sadness and anger, and heightens the risk of depression. As posited by the Need-Threat Temporal Model, individuals go through three stages after experiencing social exclusion: reflexion, reflection, and retreat. The reflection stage is crucial for assessing attributions and initiating protective behaviors (Chen C. et al., 2024). Ren et al. (2016) observed that individuals often cope with exclusion through prosocial, antisocial, or withdrawal behaviors. Nevertheless, compensatory consumption has been identified as an important strategy for alleviating the adverse effects of social exclusion. Drawing on Wang and Tian’s (2025) conceptualization, compensatory consumption serves as a substitute mechanism to compensate for psychological deficits or threats through purchasing behavior. When individuals face external threats such as social exclusion, they may engage in compensatory consumption to maintain self-integrity.

As PDE is conceptualized as a contemporary form of traditional social exclusion manifested in the information age (Van Dijk, 2020), it is reasonable to expect that PDE will elicit similar compensatory responses. Although prior research has established the link between social exclusion and compensatory consumption (Chen C. et al., 2024; Ren et al., 2016; Wang and Tian, 2025), the specific relationship between PDE—particularly in tourism contexts—and CCB among older adults remains untested. Based on this reasoning, the following hypothesis is proposed:

*H1*: Perceived digital exclusion is positively associated with compensatory consumption behavior.

### 2.2 The mediating role of perceived loss of control

Sense of control refers to an individual’s belief in their capacity to predict, explain, influence, and alter the occurrence and development of events (Raines et al., 2014). Humans have an innate desire for control, which has played a vital role in survival and evolution. However, in many contexts—such as when experiencing negative events—individuals often perceive a reduced ability to exert control over their environment, leading to perceived loss of control (PLC). According to Newcomb and Harlow (1986), PLC specifically denotes the perception that one lacks influence and control over external events.

As noted earlier, social exclusion threatens four fundamental human needs, including control (Chen J. W. et al., 2024). PDE is considered a new form of traditional social exclusion manifested in the information age (Van Dijk, 2020). Moreover, in tourism settings, older adults encounter multidimensional exclusion encompassing digital barriers, social relationships, and service facility accessibility (Seifert et al., 2021). When older tourists face exclusion due to their inability to effectively use digital technologies (Chen S. X. et al., 2024), this experience may threaten their perceived control over the travel environment. Accordingly, PDE is likely to be associated with PLC among OTs, leading to the following hypothesis:

*H2*: Perceived digital exclusion is positively associated with perceived loss of control among older tourists.

Compensatory control theory posits that the psychological significance of control lies not only in fulfilling one’s sense of agency and competence, but also in counteracting psychological discomfort caused by environmental uncertainty (Raines et al., 2014). When personal control is threatened, internal defense mechanisms are activated, prompting individuals to adopt proactive coping strategies—such as enhancing subjective agency, aligning with powerful external control systems, or affirming the consistency of behavioral outcomes—to preserve internal order and stability (Landau et al., 2015). In consumer behavior research, this logic has received empirical support: individuals who experience a loss of control tend to seek compensatory consumption as a means of restoring their sense of agency and control (Wang and Tian, 2025). Ma (2025) further suggested that individuals address control needs specifically through compensatory consumption rather than through other forms of consumption. Thus, when social exclusion threatens consumers’ control needs, they exhibit an increased preference for compensatory consumption.

Building on this reasoning, this study posits that when OTs face exclusion from digital technology, they tend to experience a reduced sense of control, which may generate psychological insecurity. To restore or compensate for this PLC, OTs may engage in consumption behaviors as a way of seeking psychological comfort and reaffirming self-worth, thereby showing a propensity for CCB. Accordingly, this study proposes the following hypotheses:

*H3*: Perceived loss of control is positively associated with compensatory consumption behavior among older tourists.

*H4*: Perceived loss of control mediates the relationship between perceived digital exclusion and compensatory consumption behavior.

### 2.3 The moderating role of self-affirmation

Steele and Liu (1983) found that because sources of self-integrity are substitutable, individuals can compensate for failure in one domain by affirming their successes in other domains. Consumption has been identified as an important means of maintaining self-integrity (Sivanathan and Pettit, 2010). Self-affirmation (SA), as a key mechanism for restoring self-integrity, reduces consumption behaviors that arise when individuals face external threats to their self-integrity—a finding supported by multiple studies (Sivanathan and Pettit, 2010). Sherman and Cohen (2006) further noted that SA has dual effects: it sustains psychological stability through the reallocation of cognitive resources, while also promoting adaptive change by leveraging values across different domains. It can therefore be inferred that OTs experiencing PDE may reduce CCB through SA.

Previous studies have shown that when an individual’s sense of control is threatened, engaging in direct SA can help restore that sense of control (Whitson and Galinsky, 2008). Additionally, Khoo et al. (2024) provided empirical evidence that administering SA to individuals mitigates the need for control triggered by threats to their sense of agency. Drawing on the substitutability principle of self-integrity (Steele and Liu, 1983) and the established link between SA and reduced compensatory responses (Sivanathan and Pettit, 2010), it can be inferred that after OTs experiencing PLC undergo SA, the control demand arising from threatened control perception is inhibited, leading to a significant reduction in CCB. Based on this reasoning, the present study proposes the following two hypotheses:

*H5*: Self-affirmation negatively moderates the relationship between perceived digital exclusion and compensatory consumption behavior among older tourists.

*H6*: Self-affirmation negatively moderates the positive relationship between perceived loss of control and compensatory consumption behavior among older tourists.

### 2.4 Social cognitive theory

The SCT originated from Bandura’s work in the 1970s, framing individual action as the product of dynamic interactions among environmental factors, cognitive processes, and behavioral patterns. This triadic framework—often referred to as a “cognition–behavior–environment” model—has been widely adopted for analyzing human behavior (Cai and Shi, 2022). Environmental influences in this framework extend beyond individual attributes to include both physical conditions (e.g., equipment and resource availability) and social surroundings (e.g., family, friends, colleagues, and relational contexts) (Bandura, 1977). Key contributions of the theory lie in its treatment of observational learning, self-efficacy, and reciprocal determinism. Research has shown that individuals can acquire new behaviors through observing others and the consequences that follow; reinforcement of a behavior—whether positive or negative—increases its likelihood of being replicated, while punishment diminishes that likelihood (Nickerson, 2024).

Early applications of SCT in consumer research focused largely on offline contexts, such as repurchase decisions and sustainable consumption (Phipps et al., 2013; Kursan Milaković, 2021). Its scope later broadened to include tourism and hospitality (Wang and Guo, 2025). Within tourism scholarship, the theory has been mobilized to explain consumer responses across a range of scenarios. These include the role of outcome expectations in shaping intentions to use travel apps in rural tourism (Lu et al., 2015), the motivational drivers of sustainable tourism (Font et al., 2016), the influence of technological service innovation on revisit behavior in digital tourism settings (Preko et al., 2023), and the effects of technostress on visitor satisfaction in hotel contexts (Lee et al., 2023). More recently, scholars have extended its use to examine children’s learning experiences during family travel (Li et al., 2024) and the pathways through which eco-friendly hotels shape green patronage and word-of-mouth (Nosrati et al., 2025). Across these studies, SCT has consistently demonstrated its value in predicting consumer decision-making.

While alternative frameworks such as the Technology Acceptance Model or Theory of Planned Behavior focus primarily on individual attitudes or perceived usefulness, they are less equipped to capture the reciprocal interplay between environmental constraints, cognitive appraisals, and behavioral responses. SCT offers three distinct advantages. First, its principle of reciprocal determinism explicitly recognizes that behavior emerges from the ongoing interaction between environment (PDE), cognition (PLC), and action (CCB)—aligning with the iterative nature of older tourists’ digital encounters. Second, SCT provides a theoretical foundation for PLC as a cognitive mechanism, transforming what could otherwise appear as a simple mediation model into an explanation of why external exclusion translates into internal psychological states and subsequent behavior. Third, SCT’s emphasis on self-efficacy and agency naturally accommodates SA as a moderator, as both constructs concern individuals’ capacity to maintain self-integrity under threat. Thus, SCT does not merely label the mediation-moderation structure; it explains the underlying psychological dynamics that give the model its coherence.

**Figure 1:**
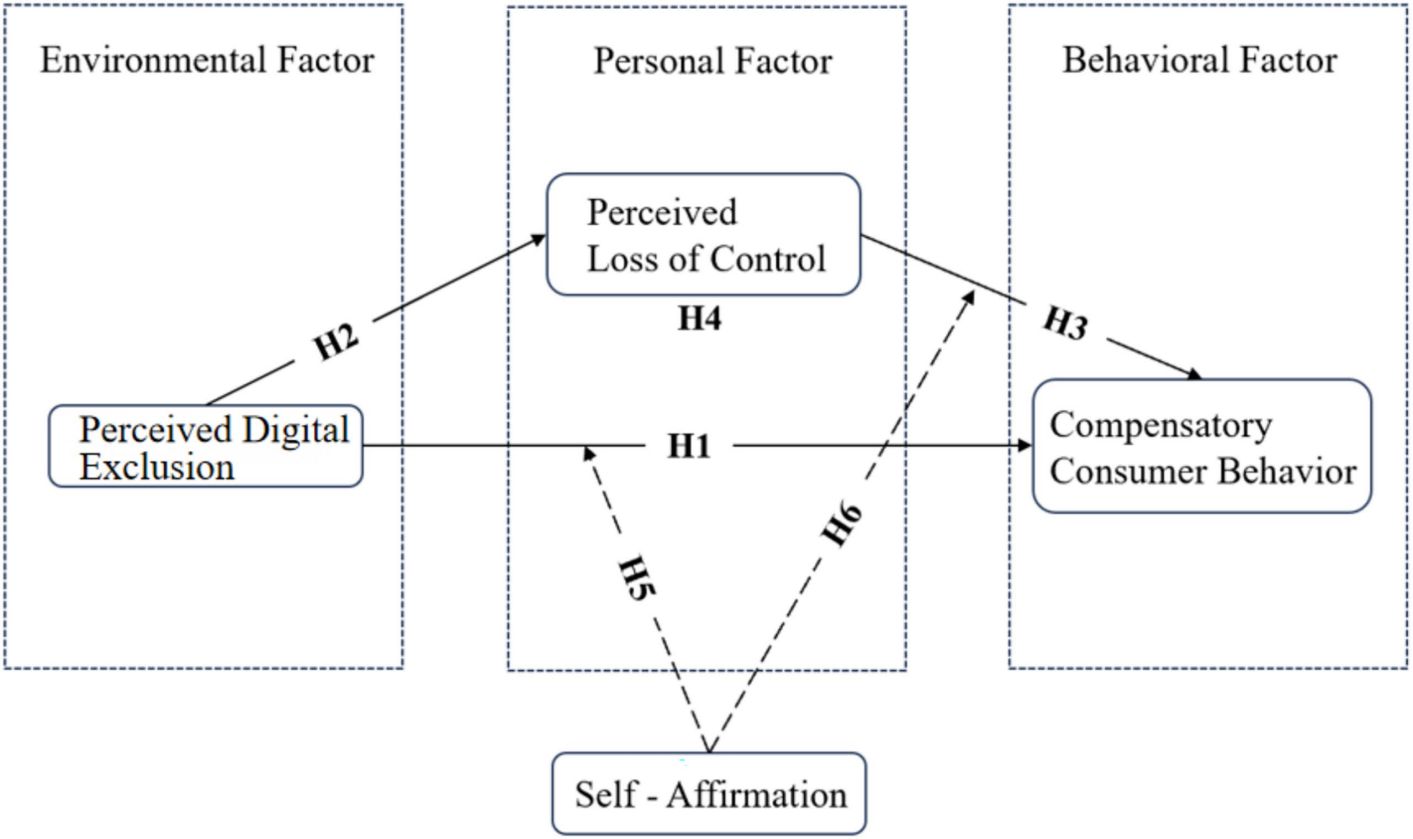
Research model.

Figure 1 illustrates the aforementioned hypotheses. The model was constructed within the scope of SCT. Kursan Milaković (2021) triadic reciprocal determinism posits that the social environment (e.g., PDE), individual cognition (e.g., PLC), and behavioral performance (e.g., CCB) form a dynamic and mutually constitutive system. Among these, PLC is defined as an individual’s belief in their inability to influence external events (Newcomb and Harlow, 1986), a perception that becomes especially prominent in contexts of PDE (Van Dijk, 2020). According to compensatory control theory (Landau et al., 2015), a perceived lack of control elicits compensatory behaviors, and consumption represents one of the primary strategies for restoring a sense of control (Ma, 2025).

## 3 Method

### 3.1 Instrument

The scales used in this study underwent rigorous validation before being adapted and applied. The questionnaire consisted of two main sections: basic information and measurement scales. The basic information section collected demographic variables including gender, household registration, age, and monthly income, and included screening questions to ensure that respondents had traveled within the past 3 months. For the measurement scales, a seven-point Likert scale (1 = strongly disagree, 7 = strongly agree) was used to assess perceived digital exclusion (PDE) and compensatory consumption behavior (CCB) among older adults in tourism settings.

The CCB scale was based on the dimension and item framework developed by Wang L. et al. (2023) and Wang Q. et al. (2023), comprising four items designed to capture behaviors such as opting for offline services and preferring physical consumption (e.g., “When travel applications are difficult to use, offline tour guide services can be purchased to improve the travel experience”). The PLC scale was adapted from the structure established by Pearlin and Schooler (1978), using five items to measure the perceived lack of control resulting from digital technology barriers (e.g., “The interface design of the mobile application is not adapted to the usage habits of the elderly, which leads to increased operational errors on my part and leaves me feeling overwhelmed”). The SA scale drew on the work of Harris and Napper (2005), retaining five items to evaluate an individual’s capacity for self-affirmation when confronted with technical difficulties (e.g., “When faced with technical challenges during travel, I do not consider my self-worth to be diminished by such experiences”). All scale items were semantically simplified and contextualized—for instance, by supplementing plain-language explanations—in accordance with the cognitive characteristics of the elderly population, thereby ensuring the applicability and content validity of the measurement instrument.

It is worth noting that not all instances of opting for offline services or seeking assistance necessarily reflect compensatory consumption; such behaviors may sometimes represent practical adaptation to situational constraints. In this study, we focus specifically on consumption behaviors driven by the psychological need to restore self-worth or control following experiences of PDE, consistent with the theoretical definition of compensatory consumption (Wang and Tian, 2025). Thus, the measurement items were designed to capture consumption responses motivated by psychological compensation rather than purely utilitarian problem-solving.

Perceived Digital Exclusion (PDE) Scale. The measurement of PDE follows the three-level framework of digital exclusion—digital access, digital use, and digital confidence/skills (Wilson-Menzfeld et al., 2025). Items were adapted from Ragu-Nathan et al.’s (2008) technostress scale, which we interpret as capturing PDE in tourism contexts: frustration and difficulty in using digital tools reflect a mismatch between system demands and individual capabilities, which constitutes the essence of PDE (Van Dijk, 2020; Chen S. X. et al., 2024; Yao et al., 2025). The five retained items map onto the three levels as follows:

Digital access: items addressing availability and usability (e.g., “The complexity of using mobile travel apps to book hotels or tickets makes me feel frustrated”).

Digital use: items concerning actual operation during travel (e.g., “I find it difficult to use electronic guide devices at scenic spots”).

Digital confidence/skills: items assessing perceived competence and self-efficacy (e.g., “I feel anxious when I have to complete a payment via mobile app while traveling”).

This operationalization ensures PDE is measured as a multidimensional construct consistent with contemporary digital divide literature. All items were semantically simplified and contextualized to suit the cognitive characteristics of the elderly population.

### 3.2 Sampling and data collection

This study employed a questionnaire-based survey to collect data from older tourists aged 60 and above. Given the characteristics of the target population—including limited geographic concentration, variability in digital literacy, and the need for face-to-face assistance during survey completion—a non-probability sampling approach combining convenience and purposive sampling was adopted. This strategy is widely recognized as appropriate for studies involving hard-to-reach or vulnerable populations, such as older adults, where probability sampling may be impractical due to low response rates, high costs, or difficulties in accessing comprehensive sampling frames (Etikan et al., 2016; Hair et al., 2014). Purposive sampling also ensured that respondents met the key inclusion criteria—age 60 or above and travel experience within the past 3 months—thereby enhancing the relevance and validity of the collected data.

In the pilot phase, the research team pre-tested the questionnaire in community senior centers and parks, distributing 153 questionnaires and receiving 122 valid responses. Based on reliability and validity tests of the pilot data, along with expert recommendations, poorly performing or ambiguous measurement items were revised or removed to improve the quality of the formal survey instrument.

During the formal survey phase, respondents were recruited through multiple community-based channels across cities in Sichuan, Henan, and Zhejiang provinces, including community senior centers, parks, and public squares. Research assistants approached potential participants in these locations, screened for eligibility (age 60 or above and travel experience within the past 3 months), and invited them to participate. To ensure geographic diversity, recruitment was conducted in both urban and suburban areas within each province. All respondents were asked to recall their most recent travel experience from the past 3 months rather than being surveyed while traveling, as this approach allowed for more efficient data collection while maintaining the temporal proximity of the recalled experience. The three-month criterion was adopted to minimize recall bias while ensuring a sufficient pool of eligible participants.

Considering common challenges among the elderly population—such as literacy barriers, visual impairments, and difficulties using electronic devices—the study adhered strictly to the principle of “assisting in completion without providing answers” to ensure both data validity and ethical compliance. All research assistants received standardized training on how to provide assistance without leading respondents’ answers. While this assisted completion approach may introduce some degree of social desirability bias or response acquiescence, it was deemed necessary to include older adults with limited literacy or vision, who would otherwise be excluded from the survey and whose perspectives are central to this study. Data collection was conducted by trained volunteers. A total of 373 questionnaires were distributed, and 328 valid responses were obtained. Respondents were mainly from cities in Sichuan, Henan, and Zhejiang provinces. Among the participants, 141 were male (43.0%) and 187 were female (57.0%). The overall effective response rate was 87.9%.

Regarding the adequacy of the sample size, the study refers to the formula for determining sample size in an infinite population proposed by Cochran (1977):


$$n_{0}=\frac{Z^{2}*p*(1-p)}{e^{2}}$$

where *Z* is the *Z*-value corresponding to the desired confidence level (*Z* = 2.576 for 99% confidence), *p* is the estimated proportion of the population (taken as 0.5 to maximize sample size), and *e* is the desired margin of error (set at 0.05). This calculation yields a recommended sample size of approximately 265. In addition, although partial least squares structural equation modeling (PLS-SEM) is capable of producing accurate and reliable results even with relatively small samples, the use of a sufficient sample size improves reliability by reducing sampling error to an acceptable level (Becker et al., 2023). Accordingly, following the recommendation of Sarstedt et al. (2022), GPower was employed as an alternative approach to determine the required sample size (Faul et al., 2009). The analysis conducted with GPower indicated that a minimum sample size of 155 is necessary (*f*^2^ = 0.15, *α* = 0.01, power = 0.9). The final sample of 328 valid responses exceeds both the Cochran formula recommendation and the G*Power threshold, confirming that the sample size is sufficient for the analyses conducted in this study.

## 4 Results

### 4.1 Demographic analysis of the sample

Male participants (43.0%, *n* = 141) were slightly outnumbered by females (*n* = 187). Regarding age distribution, the highest proportion was observed among those aged 60–65, accounting for 35.1%. Of the participants, 51.5% were from urban areas and 48.5% from rural areas, indicating a relatively balanced distribution.

### 4.2 Data analysis

#### 4.2.1 Evaluation of measurement model

This study adopted the partial least squares (PLS) estimation method, and data analysis was conducted using SmartPLS 4.0 software. Reliability was assessed primarily through Cronbach’s α and composite reliability (CR). As shown in Table 1, the Cronbach’s α values for the three latent variables—PDE, CCB, and PLC—all exceeded the conventional threshold of 0.7, and all composite reliability values were above 0.7, indicating good internal consistency.

**Table 1:** Measurement model results.

Construct	Item	Loading	Cronbach‘s α	CR	AVE
Perceived Digital Exclusion (PDE)	DE1	0.596	0.821	0.862	0.59
	DE2	0.84			
	DE3	0.831			
	DE4	0.768			
	DE5	0.846			
Perceived Loss of Control (PLC)	LPC1	0.844	0.885	0.886	0.685
	LPC2	0.79			
	LPC3	0.834			
	LPC4	0.812			
	LPC5	0.857			
Compensatory Consumption Behavior (CCB)	CCB1	0.865	0.842	0.845	0.679
	CCB2	0.802			
	CCB3	0.807			
	CCB4	0.821			

All factor loadings are significant at *p* < 0.001.

Validity assessment included both convergent and discriminant validity. The average variance extracted (AVE) for the three latent variables—PDE, CCB, and PLC—was greater than 0.5, confirming adequate convergent validity (Hair et al., 2014). For the PDE scale, one item (PDE1) had a factor loading of 0.59. While this falls slightly below the commonly suggested threshold of 0.70, it still exceeds the minimum acceptable level of 0.50 proposed by Chin (1998) for exploratory research. Given that this item contributes to content coverage of the PDE construct—particularly in capturing perceived complexity in mobile payment scenarios—it was retained. The remaining PDE items all exceeded 0.70, and both reliability and convergent validity for the PDE construct remained well above the recommended thresholds after retention.

Discriminant validity was evaluated using the Fornell–Larcker criterion (Fornell and Larcker, 1981). As shown in Table 2, the diagonal values exceed the corresponding off-diagonal values in their respective columns, indicating satisfactory discriminant validity. In addition, we assessed discriminant validity using the heterotrait–monotrait (HTMT) ratio of correlations. As shown in Table 3, all HTMT values were below the conservative threshold of 0.85 (Henseler et al., 2015), further confirming that discriminant validity is adequately established among the constructs.

**Table 2:** Discriminant validity (Fornell–Larcker criterion).

Discriminant validity analysis	CCB	PDE	LPC
CCB	**0.824**		
PDE	0.605	**0.768**	
LPC	0.656	0.772	**0.828**

Diagonal values (in bold) represent the square root of the AVE; off-diagonal values represent correlations between constructs.

**Table 3:** HTMT ratios.

Heterotrait-Monotrait Ratio (HTMT) assessment for Discriminant Validity	CCB	PDE	LPC
CCB	**—**		
PDE	0.689	**—**	
LPC	0.759	0.78	**—**

#### 4.2.2 Evaluation of structure model

**Figure 2:**
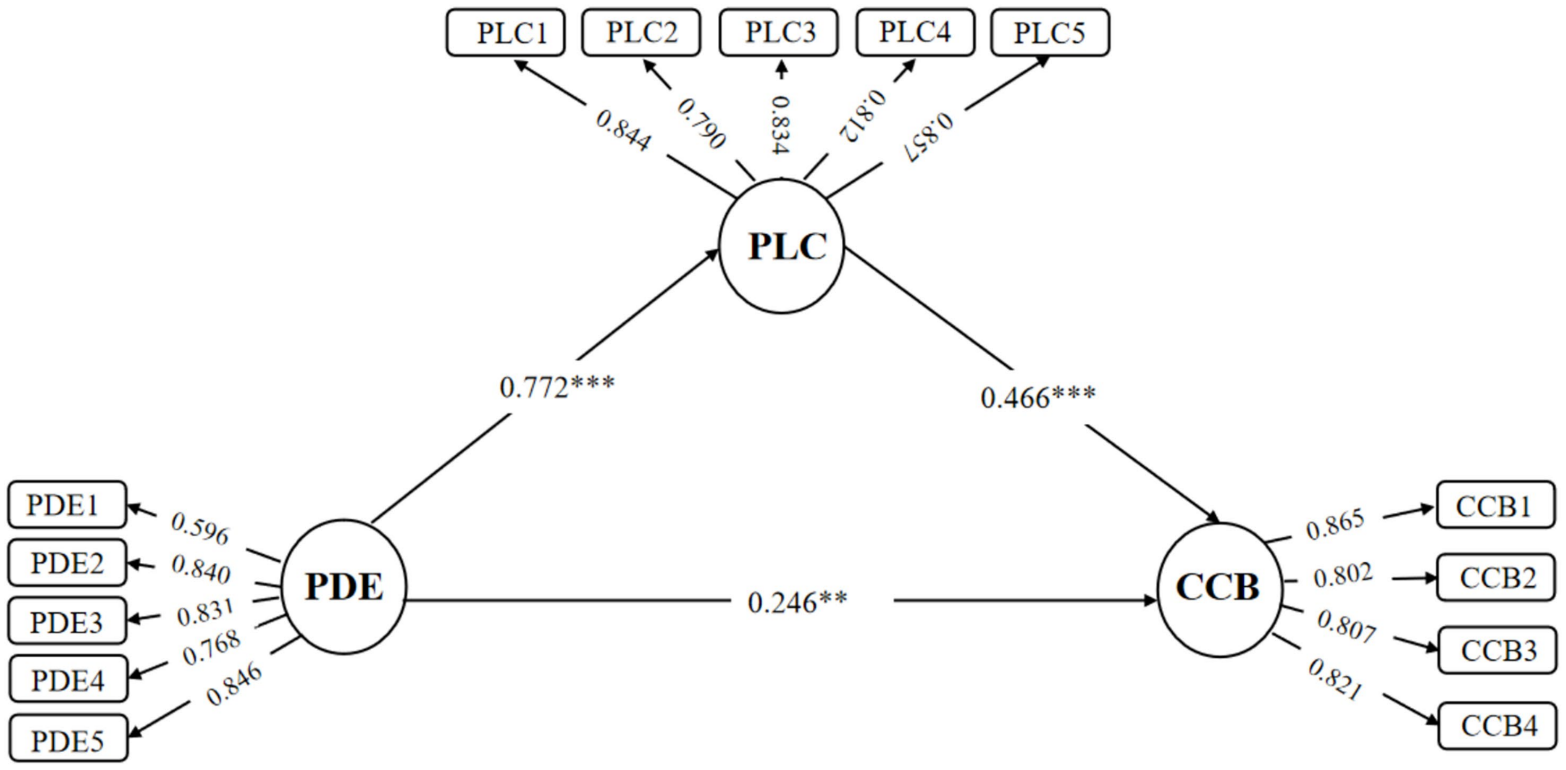
Results of the proposed model.

The structural model was evaluated using SmartPLS 4.0 with a bootstrap procedure of 5,000 samples (Esposito Vinzi et al., 2010). Evaluation criteria included the significance of path coefficients, effect size (*f*^2^), coefficient of determination (*R*^2^), predictive relevance (*Q*^2^), and goodness of fit (GOF) (Hair et al., 2014). As shown in Figure 2, all three path coefficients (0.772, 0.246, and 0.466) were significant (*p* < 0.01 or *p* < 0.001). The *R*^2^ values were 0.595 for PLC and 0.454 for CCB, indicating strong explanatory power. The Stone–Geisser *Q*^2^ values (cross-validated redundancy: *Q*^2^_PLC_ = 0.404, *Q*^2^_CCB_ = 0.304) exceeded zero, demonstrating adequate predictive relevance (Urbach and Ahlemann, 2010). According to Cohen (2013), *f*^2^ values of 0.02, 0.15, and 0.35 represent small, medium, and large effects, respectively; all values in this study met the recommended thresholds, confirming the suitability of the model.

Model fit was assessed using confirmatory factor indices, including SRMR, NFI, and RMS-theta. The overall fit indices were as follows: SRMR = 0.062 (<0.08), NFI = 0.923 (>0.90), and RMS-theta = 0.124 (<0.12), indicating acceptable model fit (Hair et al., 2019). Additionally, the GOF value was 0.622, exceeding the threshold of 0.36 proposed by Wetzels et al. (2009), demonstrating that the research model exhibits high overall quality.

#### 4.2.3 Multicollinearity and common method bias

Harman’s single-factor exploratory factor analysis is widely used to detect common method bias (CMB) (Fuller et al., 2016). In this study, a single unrotated factor explained 36.25% of the variance, below the 50% threshold, suggesting that CMB is not a significant concern. Multicollinearity was assessed using the variance inflation factor (VIF), with values below the recommended threshold of 3.3 (Wong, 2013), indicating no serious multicollinearity issues.

#### 4.2.4 Testing of hypotheses

Path analysis was conducted on the latent and observed variables of the overall model, yielding validation results for each research hypothesis (Table 4). As shown in Table 4, hypotheses H1 (*β* = 0.246, SD = 0.092, *p* < 0.01), H2 (*β* = 0.772, SD = 0.032, *p* < 0.001), and H3 (*β* = 0.466, SD = 0.088, *p* < 0.001) were all supported.

**Table 4:** Analysis of model path coefficients.

Hypotheses	Path	SD	Coefficient	*t*-value	*p*-value	Supported
H1	PDE → CCB	0.092	0.246	2.677	**	**Y**
H2	PDE → PLC	0.032	0.772	24.394	***	**Y**
H3	PLC → CCB	0.088	0.466	5.290	***	**Y**

*P < 0.05, **P < 0.01, ***P < 0.001.

#### 4.2.5 Testing of mediating effects

Mediation effects were tested using the bootstrap method, a nonparametric resampling procedure that does not require distributional assumptions and is suitable for non-normally distributed mediation effects. Following the bootstrap-based multiple mediation analysis procedure described by Zhang and Kang (2016), 1,000 repeated samples were drawn with replacement at a 95% confidence level. As presented in Table 5, the indirect effect of PLC was 0.36 (*t* = 5.152, *p* < 0.001), with a 95% bootstrap confidence interval ranging from 0.220 to 0.494. Since this interval does not include zero, PLC mediates the relationship between PDE and CCB, thus supporting Hypothesis H4.

**Table 5:** Mediation effect test.

Hypotheses	Path	Coefficient	SD	*T*-value	95% Confidence interval	Supported
H4	PDE → PLC → CCB	0.36	0.07	5.152***	[0.22, 0.494]	**Y**

*P < 0.05, **P < 0.01, ***P < 0.001.

#### 4.2.6 Testing of moderating effects

The moderation effects were tested using hierarchical linear regression in SPSS rather than within the PLS-SEM framework for two reasons. First, the study involves a single moderator (self-affirmation) interacting with two independent variables (PDE and PLC). Hierarchical regression with mean-centering and product terms is a widely accepted approach for testing such interactions and allows for straightforward interpretation of incremental variance explained (Δ*R*^2^) (Aiken, 1991). Second, given that the moderation effects were not the central focus of the structural model, this approach provides a more parsimonious analysis without overcomplicating the PLS path model.

We used SPSS 29.0 to perform hierarchical linear regression. In Step 1, control variables, PDE, and PLC were included in the regression equation (Model 1). In Step 2, the moderator SA and the interaction terms (PDE × SA and PLC × SA) were added (Model 2). To reduce potential multicollinearity, all variables used in product terms were mean-centered. The results are presented in Table 6.

**Table 6:** Regulating effect test.

Variable	Dependent variable: compensatory consumer behavior		Dependent variable: compensatory consumer behavior	
	Model 1	Model 2	Model 1	Model 2
Gender	0.112	0.101	0.112	0.101
Age	0.024	0.020	0.024	0.020
Region	0.006	−0.033	0.006	−0.033
Perceived Digital Exclusion	0.180**	0.158*	0.180**	0.158*
Perceived Loss of Control	0.522***	0.507***	0.522***	0.507***
Perceived Digital Exclusion * Self-Affirmation		−0.101*		
Perceived Loss of Control * Self-Affirmation				−0.089*
*R* ^2^	0.444	0.453	0.444	0.452
*F*	130.003***	89.601***	130.003***	88.931***
ΔR		0.009		0.008
D-W		1.596		1.592

*P < 0.05, **P < 0.01, ***P < 0.001.

As shown in Table 6, Model 1 indicates that, after controlling for demographic variables, PDE has a significant positive association with CCB (*β* = 0.180, *p* < 0.01), supporting H1. Similarly, PLC shows a significant positive association with CCB (*β* = 0.522, *p* < 0.01), supporting H3.

In Model 2, the interaction term PDE × SA has a significant negative effect on CCB (*β* = −0.101, *p* < 0.05), indicating that SA weakens the positive relationship between PDE and CCB, thus supporting H5. Likewise, the interaction term PLC × SA has a significant negative effect on CCB (*β* = −0.089, *p* < 0.05), supporting H6.

#### 4.2.7 Robustness test

To assess robustness, we additionally tested the moderation effects using the product indicator approach in SmartPLS. The results were consistent: the interaction terms remained significant (PDE × SA: *β* = −0.094, *p* < 0.05; PLC × SA: *β* = −0.083, *p* < 0.05), with comparable coefficient magnitudes, confirming that the findings are robust across analytical methods.

## 5 Conclusion and discussion

### 5.1 Conclusion

This study is grounded in social cognitive theory (Kursan Milaković, 2021) and compensatory consumption theory (Wang and Tian, 2025). Based on an empirical analysis of 328 older tourists aged 60 and above, the mechanism through which perceived digital exclusion (PDE) influences compensatory consumption behavior (CCB) was examined. The results indicate that older tourists experiencing PDE tend to report both perceived loss of control (PLC) and CCB. Compared to traditional social exclusion, PDE is more likely to trigger irrational consumption patterns (Wang L. et al., 2023; Wang Q. et al., 2023), likely due to its heavy reliance on technology and the high cost of recovery. Furthermore, PLC is positively associated with CCB among older tourists, supporting its mediating role in the PDE–CCB relationship.

In recent years, researchers from disciplines such as psychology and sociology have increasingly focused on PDE among older adults. Although research in this area has grown considerably, most studies have concentrated on antecedents rather than downstream behavioral consequences. To our knowledge, this study is among the first to link PDE with CCB, offering substantial theoretical value.

The moderating role of self-affirmation (SA) has previously been associated with negative emotions (Chen J. W. et al., 2024). This study extends that line of inquiry to the context of PDE. The findings show that when older tourists engage in SA, the positive relationship between PDE and CCB is weakened, and the positive relationship between PLC and CCB is similarly attenuated. To the best of our knowledge, this research is the first to apply social cognitive theory to examine whether and how PDE elicits CCB among older tourists, thereby making a significant academic contribution. Detailed theoretical and practical implications are discussed in the following sections.

### 5.2 Theoretical implications

This study offers three primary theoretical contributions to the literature on perceived digital exclusion and compensatory consumption in senior tourism.

First, this study extends social cognitive theory (SCT) to the context of perceived digital exclusion among older tourists. Previous research has largely relied on either internal determinism (focusing on individual traits) or external determinism (emphasizing environmental constraints) to explain older adults’ technology-related behaviors (Gallistl et al., 2023). While these perspectives offer partial explanations, they fail to capture the interactive nature of human behavior. SCT addresses this limitation by conceptualizing human activity as a dynamic interplay among environment, cognition, and behavior (Savari et al., 2024). In our framework, perceived digital exclusion serves as the environmental stimulus, perceived loss of control represents the cognitive evaluation, and compensatory consumption behavior emerges as the behavioral response. By applying SCT in this novel context, we demonstrate how external technological barriers translate into specific consumption patterns through psychological mechanisms—an application not previously examined in senior tourism research.

Second, this study identifies perceived loss of control as a key psychological mechanism linking perceived digital exclusion to compensatory consumption behavior, thereby refining the theoretical understanding of compensatory consumption among marginalized consumer populations. Existing research has established that productive goods can exert self-compensatory effects (Amos et al., 2014; Koles et al., 2018), yet the specific pathways through which technological exclusion triggers compensatory responses remain underexplored. Our findings reveal that when older tourists encounter systematic barriers in using digital technologies—a phenomenon increasingly salient in the context of smart tourism infrastructure—their sense of control is threatened, motivating compensatory consumption as a means of restoring perceived agency. This mechanism differs from previously documented compensatory pathways (Leotti et al., 2010; Pettit and Sivanathan, 2011) in that the source of control deprivation is neither purely internal nor purely situational, but rather emerges from the interaction between technological environments and age-related cognitive constraints. Thus, we provide new empirical evidence for the “PDE–CCB” framework proposed by Zhang (2013) while specifying its underlying psychological processes.

Third, this study uncovers the buffering role of self-affirmation in mitigating the effects of both perceived digital exclusion and perceived loss of control on compensatory consumption behavior. Prior research has primarily focused on how individuals respond to control threats through product preferences (Lisjak et al., 2015) or behavioral strategies (Chen J. W. et al., 2024), with less attention given to factors that can interrupt this compensatory process. Drawing on psychological immune system theory, we demonstrate that self-affirmation operates as a cognitive intervention that restores self-worth and enhances self-evaluation when older tourists face perceived digital exclusion. By replenishing internal psychological resources, self-affirmation reduces the anxiety and unease that typically drive compensatory consumption. This finding contributes to the compensatory consumption literature by identifying a boundary condition that weakens the exclusion–compensation link—an insight with implications for supporting vulnerable consumer groups in increasingly digitalized service environments.

Collectively, these contributions advance theoretical understanding of how technological environments shape consumer behavior among older populations, while opening new avenues for research at the intersection of digital inclusion, consumer psychology, and tourism management.

### 5.3 Practical implications

The findings of this study offer multidimensional practical implications for optimizing tourism service systems, formulating intergenerational digital inclusion policies, and tapping into the senior consumer market.

First, a human-centered restructuring of tourism service design is recommended. Tourism businesses should adopt a digitally inclusive service framework that addresses the needs of all visitors. For instance, in the development of smart scenic areas, it is essential to retain manual service counters that provide non-digital alternatives—such as physical information desks—for core functions including booking, ticketing, and guided tours. Such measures can help reduce the perceived loss of control (PLC) often experienced by older tourists (OTs) due to technological barriers. Destination managers are also encouraged to design control-enhancing tourism products, such as skill-certification programs (e.g., traditional craft workshops or local cooking classes), which can empower tourists and reinforce their sense of autonomy. These tangible experiences help OTs rebuild a sense of competence, aligning with their psychological tendency to regain control through familiar domains. In addition, service touchpoints can be optimized by integrating a “progressive guidance” feature into digital interfaces: when hesitation is detected in older users’ operations, voice instructions can be automatically activated or the process simplified to minimize frustration.

Second, a government-led digital inclusion ecosystem should be established. Digital accessibility certification standards for the elderly should be developed within tourism contexts, requiring that all 4A-level and above scenic areas obtain barrier-free service certification, while digital inclusion metrics are integrated into the scenic area rating system. A dedicated fund for digital literacy in senior tourism could support communities in offering “smart tourism practical courses,” which enhance older adults’ ability to use digital technologies through scenario-based instruction.

Third, tourism technology companies are encouraged to develop smart terminals with “cognitive compensation” features, such as voice-enabled interactive guide devices, to reduce technical barriers through multimodal interaction. Tourism marketing organizations should craft advertising narratives that emphasize self-efficacy, portraying scenarios in which OTs successfully demonstrate personal competence through tourism consumption—for example, by using smart devices to book unique experiences. In addition, “fluid compensation” product packages can be designed to connect digital service failures with offline consumption opportunities, such as offering reservation codes for in-person activities as compensation when digital transactions encounter difficulties.

### 5.4 Limitations and future directions

Although this study yields significant findings, several limitations should be acknowledged. First, non-probability sampling methods—namely convenience and purposive sampling—were adopted, which may constrain the representativeness of the sample. While this strategy is appropriate for accessing hard-to-reach populations such as older adults, the findings may not be fully generalizable to the broader population of older tourists. Moreover, data were collected exclusively within mainland China, where cultural, technological, and tourism contexts may differ from those in other countries or regions. Therefore, generalizability beyond the Chinese context should be interpreted with caution. Future studies could employ probability sampling approaches, such as random sampling across multiple countries or regions, to improve generalizability.

Second, although questionnaires were used to accommodate the circumstances of elderly participants, this method is limited in capturing the dynamic nature of consumer behavior. In addition, assisted completion—necessary to include older adults with literacy or vision limitations—may have introduced some degree of social desirability or acquiescence bias. To mitigate this risk, research assistants were trained to provide standardized assistance without leading respondents’ answers. Future research could integrate experimental methods (e.g., simulating PDE scenarios) or longitudinal tracking to gain deeper insights into behavioral mechanisms, while also exploring alternative data collection modes such as audio-assisted digital surveys to reduce potential bias.

Third, the cross-sectional design limits causal inference regarding the mediating role of perceived loss of control. Although the mediation analysis reveals statistically significant indirect effects, these findings reflect associations rather than causal pathways (Maxwell and Cole, 2007). While the proposed mediation model is grounded in social cognitive theory—which specifies a directional relationship from environment to cognition to behavior—the cross-sectional data cannot fully establish temporal precedence. Future research could employ longitudinal designs or experimental manipulations to further test the causal ordering of the relationships examined.

Additionally, the moderating role of self-affirmation merits further exploration. For instance, while we found that SA mitigates the effect of PDE on CCB, it remains unclear whether this relationship is weakened or strengthened among older adults with high versus low levels of SA—a question that could be addressed in future research. Finally, future studies should prioritize developing strategies that guide older tourists toward healthy and positive compensatory consumption, thereby reducing the risk that they merely transition from the negative effects of PDE to more detrimental or financially burdensome consumption patterns. For older tourists who develop detrimental consumption patterns due to PDE, approaches such as consumption guidance and psychological interventions could be explored to encourage positive and healthy compensatory consumption. Such efforts would help alleviate the adverse effects of PDE and further promote digital inclusion among older tourists.

## Data Availability

The raw data supporting the conclusions of this article will be made available by the authors, without undue reservation.
